# Aflatoxin Detoxification Using Microorganisms and Enzymes

**DOI:** 10.3390/toxins13010046

**Published:** 2021-01-09

**Authors:** Yun Guan, Jia Chen, Eugenie Nepovimova, Miao Long, Wenda Wu, Kamil Kuca

**Affiliations:** 1 Key Laboratory of Zoonosis of Liaoning Province, College of Animal Science & Veterinary Medicine, Shenyang Agricultural University, Shenyang 110866, China; guanyun19950530@163.com (Y.G.); chen15524373937@163.com (J.C.); 2 Department of Chemistry, Faculty of Science, University of Hradec Kralove, 50003 Hradec Kralove, Czech Republic; eugenie.nepovimova@uhk.cz; 3 MOE Joint International Research Laboratory of Animal Health and Food Safety, College of Veterinary Medicine, Nanjing Agricultural University, Nanjing 210095, China

**Keywords:** aflatoxin, biological detoxification, detoxification mechanism, degradation products, probiotics

## Abstract

Mycotoxin contamination causes significant economic loss to food and feed industries and seriously threatens human health. Aflatoxins (AFs) are one of the most harmful mycotoxins, which are produced by *Aspergillus flavus*, *Aspergillus parasiticus*, and other fungi that are commonly found in the production and preservation of grain and feed. AFs can cause harm to animal and human health due to their toxic (carcinogenic, teratogenic, and mutagenic) effects. How to remove AF has become a major problem: biological methods cause no contamination, have high specificity, and work at high temperature, affording environmental protection. In the present research, microorganisms with detoxification effects researched in recent years are reviewed, the detoxification mechanism of microbes on AFs, the safety of degrading enzymes and reaction products formed in the degradation process, and the application of microorganisms as detoxification strategies for AFs were investigated. One of the main aims of the work is to provide a reliable reference strategy for biological detoxification of AFs.

## 1. Introduction

Mycotoxins are metabolites of fungi that are ubiquitous in cereal crops and animal forage [1]. One group of well-known mycotoxins, aflatoxins (AFs), are secondary metabolites produced mainly by *Aspergillus flavus*, which produces both aflatoxin B_1_ (AFB_1_) and aflatoxin B_2_ (AFB_2_), and by *Aspergillus parasiticus*, which produces aflatoxin G_1_ (AFG_1_) and aflatoxin G_2_ (AFG_2_) [2]. They have a high degree of hepatotoxicity, nephrotoxicity, and immunotoxicity [3]. Among them, AFB_1_ is the most toxic and is well known for its toxic carcinogenic and teratogenic mutation effects [4,5]. As a result, it was categorized as a Class I carcinogen by the World Health Organization in 1993 [6,7].

**Figure 1:**
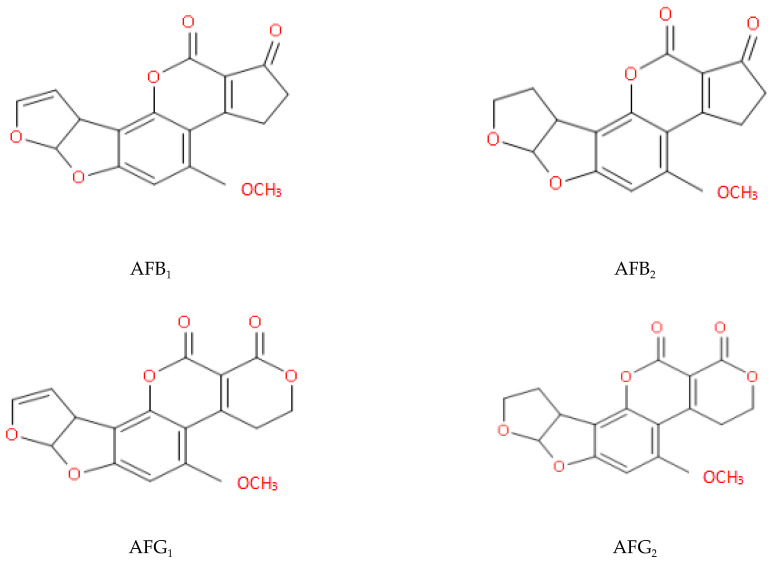
Structures of some natural AFs (Aflatoxins B_1_ and G_1_ have double bonds at positions 8–9; aflatoxins B_2_ and G_2_ do not).

The long-term consumption of food contaminated with AFs can induce inflammatory damage to hepatocytes [8]. Furthermore, the AF-DNA adducts can result in the production of cancer cells [9], leading to liver cancer [10,11]. In addition, AFB1 can induce the apoptosis of CASP3 and BAX, and shows extensive cytotoxicity to neuronal cells, including ROS accumulation, DNA damage, S-phase arrest, and apoptosis [12]. AFs can also destroy the metabolic pathways of a variety of intestinal flora. This may affect energy supply and lead to certain metabolic diseases [13,14]. Today, South-East Asia remains a high-risk area for acute AF poisoning [15]. Molecular structures of four naturally occurring AFs are illustrated in Figure 1.

AFs are often detected in grains, nuts, and spices [16,17]. Contamination occurs readily when feed and food are exposed to high temperature and high humidity [18]. The toxic effects of AFs are not only manifested in feeding. Animals that consume contaminated feed are likely to be poisoned [19]. However, the toxins found in the animal by-products (e.g., milk and milk products) will enter other animals in the food chain, which can result in further serious consequences and spread the contamination more widely [20]. Finding ways to safely and efficiently detoxify food has thus become a focus of research [21]. Contamination of AFs in food and feed samples in some countries is displayed in Table 1.

AFs can be detoxified using physical, chemical, and biological detoxification methods, and a great deal of research has been carried out using these methods in the past few decades [31,32]. Physical methods are those most commonly used; for example, adsorbents are employed to undertake physical adsorption to control toxin contamination [33]. Although adsorbent products can reduce the bioavailability of mycotoxins, in practice, the toxins cannot be completely adsorbed [34]. In recent years, after continuous improvement, nanotechnology has been applied to adsorbents, such as magnetic adsorbents, whose adsorption capacity has been much improved [35]. However, physical methods show many disadvantages, e.g., limited applicability, poor detoxification effect, and limited detox product status [36]. Chemical methods involve treatment with acid, alkali, or oxidizing agent [37]. The use of chemical substances such as chlorine dioxide to disinfect toxins [38] may impair the appearance and taste of food. After chemical treatment, chemical residues in food may be harmful to humans [39]. Neither approach is the better option for detoxification. Biological detoxification also has certain drawbacks, such as the difficulty of controlling microbial performance and the safety of the newly formed product to the body [39]; however, biological detoxification has high specificity, produces harmless products, and can even completely detoxify samples under appropriate conditions [37,40]. Thus, biological detoxification is gradually becoming the most suitable detoxification approach [41,42].

Beneficial intestinal bacteria have many important functions. They produce various nutrients for the host, prevent infections caused by intestinal pathogens, and regulate the immune response [43]. At the same time, the life activity metabolites of microorganisms (such as exogenous antioxidant compounds) can induce activity among genes related to the oxidative stress toxicity of AFs, restore the oxidative balance destroyed by mycotoxins, and prevent the production of ROS and RNS [44]. Therefore, the use of microorganisms to detoxify AFs is a promising new technology with broad application prospects; as such, their use is a research hotspot both for the beneficial effects and AF detoxification [41,45].

## 2. Microorganisms with Detoxification Effects

Different microorganisms exert detoxification effects toward AFs [46]. The microorganisms that exert detoxification effects on AFs are listed in Table 2.

## 3. Decontamination Mechanism of AFs

### 3.1. Microorganisms Inhibit the Production of AFs

Mixed populations of microorganisms coexist in the ecosystem, thus forming a complex microbial community [86]. Soil is the natural habitat of *Aspergillus flavus*, and soil ecotoxicology has gradually become a safety hotspot [87]. The high complexity and heterogeneity of the soil environment make it difficult to analyze the ecological functions of secondary metabolites such as AFs in the soil [87]. Therefore, co-cultivation research has become an effective means to control or reduce specific contaminants in grain, feed, and the environment [88].

Competitive interactions between pathogenic and beneficial microorganisms include both exploitation and interference competition [89]. When *Aspergillus flavus* and *Aspergillus parasiticus* are co-cultured with Salmonella, the colony diameter and spore formation of *Aspergillus flavus* and *Aspergillus parasiticus* are decreased, and the contents of AFs (AFB_1_, AFB_2_, AFG_1_, and AFG_2_) are reduced [86]. After 24 h of co-cultivation of *Aspergillus flavus* and *Aspergillus niger*, the growth of *Aspergillus flavus* was inhibited and the production of AFB_1_ was also reduced by 42.8% [80]. Further studies implied that, during co-cultivation, the life activities of other microorganisms can cause gene mutations or activate silent gene clusters, thereby reducing the production of AFs [90]. The biosynthetic processes that generated AFB_1_ in *Aspergillus flavus* were interrupted when the *A. flavus* was co-cultured with *Streptomyces roseolus*. More specifically, the interruption to the biosynthetic pathway occurred at an early stage before the synthesis of norsolorinic acid, so the first toxic AFB_1_ precursor could not be synthesized normally and the concentration of AFB_1_ was decreased to an undetectable level [73]. The inhibitory compounds secreted by *Aspergillus oryzae* and a non-aflatoxigenic *A. flavus* can inhibit the production of AFB_1_ and the growth and reproduction of *Aspergillus flavus*. Transcriptome sequencing has shown that some genes such as AflS, FarB, and MtfA are involved in the biosynthetic pathway of AFs. The synthetic gene cluster was significantly down-regulated, and the two conidial transcription factors BrlA and AbaA were significantly down-regulated, which may down-regulate conidia-specific genes (such as the conidial hydrophobin genes RodA and RodB) [91]. 

Toxins will exist for a long time after polluting the soil. In planting on contaminated land, toxins will be transferred from the soil to the grain, and then transferred to fodder whereafter they are accumulated. If beneficial microorganisms can multiply in the contaminated soil, the toxin content will be greatly reduced. In short, co-cultivation can indeed provide new insights for controlling the synthesis of AFs and the proliferation of *Aspergillus flavus*. The exact molecular mechanism of this process remains to be studied.

### 3.2. Microbial Adsorption of AFs

**Figure 2:**
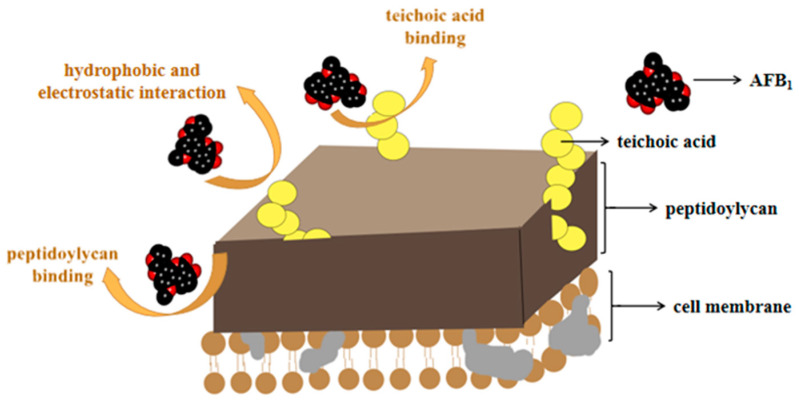
The adsorption of AFs by microorganisms (taking AFB_1_ as an example). Microorganisms can adsorb AFs through peptidoglycan or phosphoric acid in the cytoderm, and hydrophobic and electrostatic interaction.

Adsorption means that due to the special structure on the microbial cell wall, AFs interact with non-covalent bonds (the main effect is that of Van der Waals forces), which makes it easier to bind, reducing the bioavailability of mycotoxins in the gastrointestinal tract, and protecting the body from toxin infringement [92,93]. For biosorption, the most often studied strains are *Lactobacillaceae* and *Saccharomyces*, which can effectively bind AFs through polysaccharides (such as peptidoglycan and teichoic acid) on the bacterial wall [94,95]. The adsorption mechanisms thereof are illustrated in Figure 2.

*Lactobacillaceae* and *Saccharomyces* are the most commonly used microorganisms in fermentation: *Lactobacillus delbrueckii*, *Lactobacillus kefiri*, and *Lactobacillus rhamnosus strain* (LGG) are used for the fermentation of yogurt or cheese; *Saccharomyces cerevisiae* can be used for brewing beer [76,96,97]. The excellent adsorption capacity and natural fermentation function make the use of *Lactobacillaceae* and *Saccharomyces* essential in the process of detoxifying food. LGG is an excellent biosorption species. The combination of heat-treatment and anaerobic solid fermentation can remove 100% of AFB_1_ [41]. Of course, this is the result of adsorption under simulated laboratory conditions. Recent research has shown that LGG can adsorb 90% of AFs in pistachio nuts subjected to heat treatment (from an initial concentration of 20 ppb), and it had no effect on the qualitative characteristics of the pistachios, e.g., color, texture, and peroxide value [58].

Not only for food, LGG has outstanding stability with respect to stomach acid and bile, and can therefore enter the intestines of the body in vivo. It is also an excellent species to use in fermentation as it has favorable degradability (so it is safe to use during the fermentation process) and does not affect the palatability of the product [57]. It is worth noting that, although LGG is resistant to the environment in the intestine, its binding to toxins is unstable. The stability of the combination of species and toxins depends on various parameters, such as pH, temperature, sorbate ion concentration, and mixing rate [98]; therefore, careful optimization is required before application. Unlike *Lactobacillus*, however, *Saccharomyces* results in adsorption products that are more stable (i.e., less likely to re-release the toxin). The combined product forms a complex that is not readily adsorbed by the body and is mostly excreted. Hence, *Saccharomyces* species are relatively stable mycotoxin adsorbents (mainly because the toxins form a specific complementary structure with the mannose on the cell walls). A study has concluded that the adsorption capacity of *Saccharomyces* lysate with respect to AFs can reach 2.5 μg/mg [99]. The problem of how to improve the adsorption capacity of *Saccharomyces* is also a hot research topic.

In addition, *L. plantarum* not only exerts a detoxifying effect on AFs but is a biological preservative. It can inhibit the decay of animal manure and residual feed in the middle and late stages of animal breeding, reducing the amount of chemicals required and the cost of breeding. It is therefore very important in production practices [75,76].

### 3.3. Microbial Degradation of AFs

Degradation involves the microorganisms producing certain substances during their life activities that change the original structure of the mycotoxins and convert them into substances that are low in toxicity or even completely non-toxic. AFs are metabolites of difurans and the double bond in the furan ring is the main site leading to genetic mutations and carcinogenic teratogenic effects [100]. The main toxic structure present in AFs is the coumarin lactone ring, which is readily hydrolyzed [101,102,103]. During the degradation process, the active substances secreted by the microorganisms are mainly enzymes that convert the AFs into other substances. Main degrading enzymes of AFs are displayed in Table 3.

**Figure 3:**
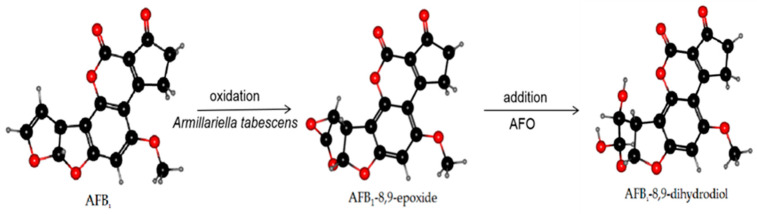
The mechanism of AFB1 degradation. *Armillariella tabescens* and the AFO produced therewith can act on the dilute ether bond of the furan ring to activate AFB_1_ transforming it into an epoxide. The hydrolysis reaction was conducted to generate a new compound with significantly reduced toxicity: AFB_1_-8,9-dihydrodiol.

AFO, as an intracellular enzyme, is a typical member of the dipeptidyl peptidase III (DPP III) enzyme family [112] and was extracted from *Armillariella tabescens*. It can act on the dilute ether bond of the furan ring of AFB1 and convert it to epoxide. Hydrolysis to generate AFB1-8,9-dihydrodiol was undertaken to achieve the purpose of detoxification [113,114]. *Armillariella tabescens* is a Chinese edible fungus, and AFO is a new choice in practical applications preventing biodegradation of food and detoxification of AF in feed. The reaction mechanism of AFO is demonstrated in Figure 3.

**Figure 4:**
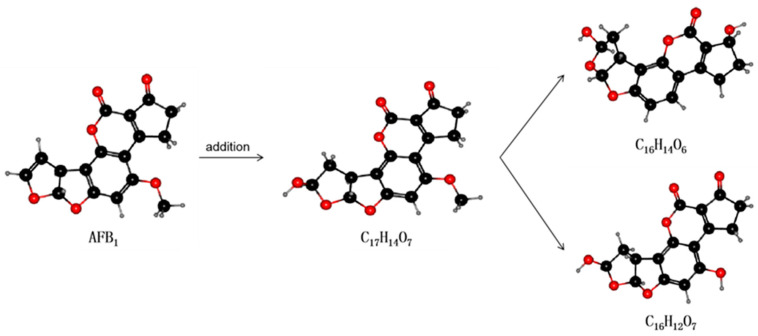
The mechanism by which laccase degrades AFB1. Laccase can act on the double bond of the furan ring to undergo an addition reaction. As shown, the degradation product with molecular formula C17H14O7 (unstable structure) is first produced, then the elimination reaction occurs to generate two degradation products with different structures: C16H14O6 and C16H12O7.

Laccase is an extracellular enzyme that contains four copper ions and can be extracted from some microorganisms (e.g., white rot fungi) [115]. Many in vitro experiments have been conducted to ascertain the stability of laccases. In vitro degradation experiments using recombinant fungal laccase found that AFB1, AFB2, AFG1, and AFG2 can interact with the laccase (near the T1 copper center of the enzyme) via hydrogen bonds and hydrophobic interactions with amino acid residues. The binding capacity of the interaction was also shown to decrease in the order AFB1 > AFG2 > AFG1 > AFB2 and the maximum degradation rates were 90.33%, 74.23%, 85.24%, and 87.58%, respectively [116]. The latest research by Zhou et al. found that a new type of laccase that catalyzes the degradation of AFB1 could be purified and identified in white-rot fungus Cerrena unicolor. The half-life of AFB1 degradation catalyzed thereby was 5.16 h, and the degradation product was AFQ1 [85]. These findings are expected to lead to the use of laccase as a new AFO able to degrade AFB1 in food and feed. The reaction mechanism of laccase is displayed in Figure 4.

There are also some newly discovered enzymes that also have detoxification capability for AFs. The alternative oxidase, which is ubiquitous in the plant kingdom, affects the penultimate intermediate of AFB1 biosynthesis [117], but after analysis of the genome sequence, alternative oxidase also has expressed genes in *A. clavatus*, *A. flavus*, *A. fumigatus*, *A. nidulans*, and *A. niger* [118]. Alternative oxidase may be used as a target to control the reproduction of *Aspergillus flavus* and contamination by AFs.

MSMEG-5998 is an AF-degrading enzyme produced by *Mycobacterium smegmatis* (*F. smegmatis*), which can reduce AFB1-induced cytotoxicity in HepG2 cells by ameliorating DNA damage and p53-mediated apoptosis. Thioredoxin affected the rate of degradation of MSMEG-5998 to AFB1 as it increased from 31% to 63% [108,119]. The MSMEG-5998 connected by thioredoxin shows great application prospects, but the toxicity of the product remains to be considered.

CotA laccase, a new aflatoxin oxidase in *Bacillus licheniformis*, can convert AF into AFQ1 and epi-AFQ1. In vitro experiments have found that AFQ1 and epi-AFQ1 do not inhibit the viability of human hepatocytes and induce apoptosis [120]. These findings are expected to allow use of CotA laccase as a new AFO to degrade AFB1 in food.

**Figure 5:**
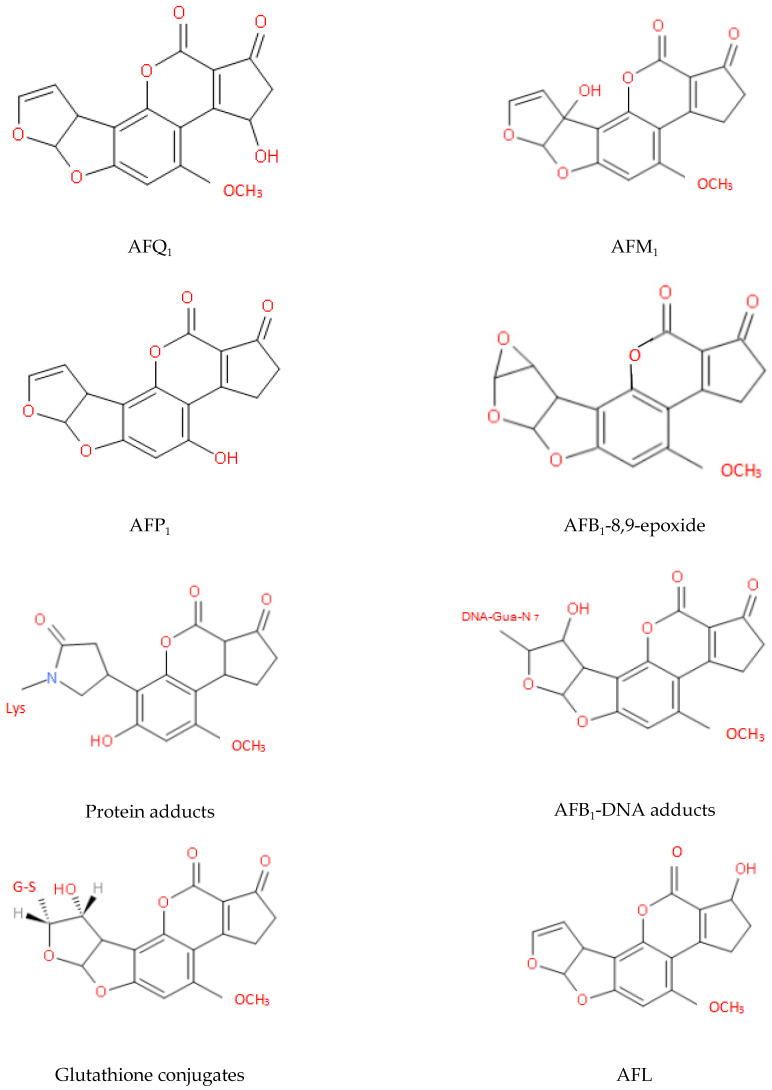
Molecular structures of some key AF metabolites.

The two key sites that affect the toxicity of AFs are the furan and lactone rings and the detoxification process mainly involves changes in the structures of these rings. After many years of research, the metabolites of AFs that have been identified fall into the following three categories: (i) hydroxylated metabolites, e.g., AFM_1_, aflatoxin P_1_ (AFP_1_), and aflatoxin Q_1_ (AFQ_1_); (ii) epoxides, e.g., AFB_1_-8,9-epoxide; and (iii) metabolites of microorganisms or animals, e.g., AFG_2a_, AFB_2a_, and aflatoxicol (AFL) [44,45,79]. The molecular structures of some of these metabolites are shown in Figure 5 [121,122,123,124].

Unlike adsorption, degradation changes the structure of toxins. The toxicity of degradation products is the most important indicator of whether degrading enzymes can be used to detoxify the body. If the degradation product is of low toxicity or even non-toxic, this degradation enzyme is applicable. On the contrary, there is no applied research value otherwise. Melvin et al. found that *Pseudomonas putida* MTCC 1274 and 2445 can tolerate AFB_1_ in the medium, break the furan and lactone rings in the AFB_1_ molecule within 24 h of incubation, and convert it into new products: a non-toxic compound, AFD_1_ and two compounds, AFD_2_ and AFD_3_, of low toxicity [66]. *Bacillus velezensis*, *Lysinibacillus fusiformis*, *Staphylococcus warneri*, and other species can also degrade AFs into new substances with significantly reduced cytotoxicity [54,125]; however, the degradation process is often accompanied by many intermediate metabolites, and it is not enough to analyze only the toxicity of the final degradation products. *Tetragenococcus halophilus* CGMCC 3792 can produce six non-toxic metabolites in the process of AFB1 degradation, and there are two completely different degradation pathways [63]. The end products of the two pathways are non-toxic C_14_H_20_O_2_ compounds [63]. The high degradation rate of AFB_1_ achieved using *T. halophilus* CGMCC 3792 and the non-toxicity of its degradation products suggest it has detoxification applications, both in vivo and in vitro, and huge application potential in the processing of fermented oriental seasonings.

AF degradation results obtained using representative microorganisms and the degradation products formed are displayed in Table 4. Separating and purifying degradation enzymes and determining the toxicity of degradation products are problems that must be faced in any clinical application of biodegradation. The degrading enzyme can be amplified and expressed according to its gene sequence, and has a good degradation effect, laying a solid foundation for its actual clinical application. The toxicity of the product is a reference indicator for the use of degrading enzymes. How to isolate degrading enzymes from a species that can degrade AFs into non-toxic metabolites will be the focus of future research.

## 4. Application of Microbial Detoxification

### 4.1. Compound Probiotics Increase the Ability to Detoxify AFs

Although many microorganisms can detoxify AFs, probiotics are the first choice for detoxification. Adding probiotics during the breeding process can help prevent AFs causing tissues lesions, especially in the liver [10]. The detoxification of AFs using probiotics often involves multiple effects; multiple species can therefore be used together to acquire a better detoxification effect. The *saccharomyces*-containing mixture present in kombucha can adsorb AFB_1_ and convert it into four products of low toxicity. Poisoning tests using brine shrimp showed that the mortality rates of these AFB_1_ degradation products were between 20% and 80%, whereas the mortality rate with AFB_1_ was up to 100% under the same conditions [128]. This result proved that this mixed yeast product can adsorb part of the toxin while converting another part into less toxic products, thus reducing the impact of AFs on cell tissues and even the body as a whole. 

Chen et al. found that *Streptococcus thermophilus* and *Lactobacillus delbrueckii* subsp. *bulgaricus* can completely remove AFB_1_ and AFG_1_ in peanuts subjected to anaerobic, high-temperature, solid fermentation conditions (to the extent that no obvious toxicity was observed in the final products) [58]. In this case, the two species facilitated excellent biotransformation under specific conditions. In general, this research was conducted under optimal growth conditions specific to the strain; however, it is necessary to ascertain the detoxification ability of strains to AFs under specific conditions.

The use of probiotics compound not only improves the rate of degradation of AFs, but also makes the intestinal epithelial barrier more resistant to mycotoxins and toxins from other pathogenic microorganisms [128]. Cavaglieri et al. showed that probiotics of certain bacteria (*Pediococcus pentosaceus* RC006) and yeasts (*Kluyveromyces marxianus* VM003) have the ability to adsorb and degrade AFM_1_ in milk to fewer toxic derivatives when used in combination [129].

The probiotic mixture used by Barati et al. (consisting of *Bacillus* and *Lactobacillus* species and cell walls of *Saccharomyces cerevisiae*) was found to reduce the inhibitory effect that AFs have on the humoral and cellular immune systems of broiler chicks. This mixture was therefore able to weaken the anti-nutritional effects of the AFs. Furthermore, it also improved the synthesis of proteins in the chicks. Thus, the mixture could control the impact of AFs on the chicks and improve their immune functions and biochemical pathways [130,131]. The combined use of probiotics to detoxify AFs in recent years is displayed in Table 5.

### 4.2. Microbial Preparations Can Remove AFs in Food and Feed

The detoxification method of AFs has attracted increasing attention; however, the in vivo detoxification reaction is difficult due to the problem of the activity of biological factors. Therefore, the in vitro detoxification study of bacterial fermentation broth is warranted. The degrading enzyme activity of *Bacillus subtilis* BCC 42005 was stable and non-toxic at IC 50.4 mg/mL. Its fermentation broth was mixed with water as a corn-soaking agent. After 2 h of contact, the content of AFB1 was decreased by 54% [136]. The 39 volatile organic compounds produced by *Streptomyces philanthi* RL-1-178 could replace toxic chemical fungicides as biological fumigants and control the production of AFB1, AFB2, and AFG2 in stored soybean seeds [137]. Therefore, microorganisms can be used as a new biological agent to reduce the contamination of AFs in food and feed.

### 4.3. Microbes Ameliorate the Damage Caused by AFs to the Body

Fan et al. researched the ability of *Bacillus subtilis* ANSB060 to detoxify AFs. Their results showed that *B. subtilis* improved the growth performance and meat quality of broilers [138]. The levels of AF residues in the livers of broilers consuming naturally moldy peanut meal were also decreased [134]. Chen et al. found that oral *Lactobacillus bulgaricus* or *Lactobacillus rhamnosus* ingestion can significantly prevent liver injury induced by AFB1, and reduce histopathological changes and inflammation by elevating the expression of NF-κB p65 [138]. Feeding with *Lactobacillus plantarum 299v* can decrease the contents of serum lactate dehydrogenase and alanine aminotransferase in the liver and increase the body weight of broilers by about 20%-55%, bringing economic benefits [139]. Therefore, microorganisms can ameliorate damage to the body induced by AFs by adjusting related pathways, or they can preferentially combine with AFs to prevent AFs from exerting their toxic effects. The oral administration of microorganisms may be a new treatment for AF poisoning.

### 4.4. Combined Use of Probiotics, Biological Agents, and Degrading Enzymes

As probiotics are safe to use and have superior detoxification ability, the combined use of compound probiotics and degrading enzymes has also been explored in recent years. For example, when a 1:1:1 mixture of *Bacillus subtilis*, *Lactobacillus casei*, and *Candida utilis* was mixed with *Aspergillus oryzae* degrading enzyme in the ratio of 3:2, the degradation rate of AFB_1_ was found to be 63.95% [135]. Another study found that using licorice extract, Protexin probiotic, toxin binder (Agrabound), and poultry litter biochar as additives, during mixed feeding of broiler chickens, can reduce the effects of AFB1 on broiler chickens, improving blood indicators, and immunity to good effect [140].

Evaluating food and feed to identify its safety will also need to be a top priority in future research. In short, the combined use of probiotics, biological agents, and degrading enzymes is another innovative strategy for mycotoxin degradation.

### 4.5. Detoxification of Mixed Mycotoxins by Microorganisms

The pollution caused by mycotoxins is often not of a single type, but of mixed types: for instance, AFs and zearalenone, etc. Beneficial microorganisms can simultaneously detoxify multiple toxins. Lactic acid bacteria have detoxification effects on AFs, Ochratoxin A, and zearalenone [141]. *B. subtilis* and *B. velezensis* have high degradation efficiency when applied to AFs and zearalenone, and the degradation products have also been studied [129]. Based on more thorough research into the mechanisms of detoxification, the joint action of multiple microorganisms and the combined use of multiple degrading enzymes will be the focus of future research.

## 5. Conclusions

The use of microorganisms (especially microorganisms with probiotic properties) is a specific, effective, environmentally friendly, cheap, and safe strategy. The pleasant harvest produced by microbial detoxification is the elimination of chemical pesticides and pollutants in food and feed, and an absence of toxic residues. At the moment, biological detoxification technology is far from perfect and the determination and purification of metabolites is incomplete in many cases. Therefore, more research is needed to reveal the mechanism, dosage, time of microbial detoxification, and how to use these new microbial preparations to maximize the prevention and beneficial effects on toxins. As the technology develops, the mechanisms by which these probiotics detoxify AFs will gradually become well known and their use as feed/food additives will be mastered and perfected.

It is, therefore, just a matter of time before the production of enzymes and microbial preparations (and other biological additives) are taken to the stage where large-scale industrialization is realized.

## Figures and Tables

**Table 1:** Contamination of AFs in food and feed.

Locality	Sample	Rate of Contamination (%)	AFs	Toxin Level ^a^	Refs
Uganda Lake Victoria Basin	Fish feed in the factory	48	B_1_	<40 µg/kg	[22]
	Fish feed in the farm	63		>400 µg/kg	
Uganda Multiple districts	Groundnut seeds	81	— ^b^	84.7 μg/kg	[23]
	Milled groundnuts			1277.5 μg/kg	
Cameroon	Catfish	100	B_1_	31.38 ± 0.29 ppb	[24]
Nigeria Ekiti State	Dried beef meat (as sold)	66	B_1_	105.4 µg/kg	[25]
			B_2_	6.92 µg/kg	
			G_1_	40.49 µg/kg	
			G_2_	2.60 µg/kg	
Mexico Mexico City	Oaxaca-type cheese (as sold)	20	B_1_	0.1 μg/kg	[26]
		30	G_1_	0.6 μg/kg	
		57	M_1_	1.7 μg/kg	
Malaysia	Raw peanuts	—	—	12.8–537.1 μg/kg	[27]
	Peanut sauce			5.1–59.5 μg/kg	
Sri Lanka Anuradhapura	Corn	63.33	B_1_	60–70 ppb	[28]
	Corn-growing soil	90		350–400 ppb	
India Mahabubnagar	Cereals in the family	82	B_1_	>1μg/ kg	[29]
Thailand	Sesame (as sold)	9	—	>2 μg/kg	[30]

a Unsigned data represent the average rate of contamination. b This symbol indicates unknown or not mentioned.

**Table 2:** Microorganisms that can be used for the detoxification of AFs.

Microorganism		Detoxification Method	Refs.
Bacillaceae	*B. velezensis*	Degradation	[47]
	*B. subtilis*	Degradation	[48,49,50,51]
	*B. pumilus*	Degradation	[52]
	*B. licheniformis*	— ^a^	[53]
Planococcaceae		Degradation	[53]
Staphylococcaceae	*S. warneri*	Degradation	[54]
Lactobacillaceae	*L. Plantarum*	Adsorption & degradation	[55]
	*L. kefiri*	Adsorption	[56]
	*L. rhamnosus*	Adsorption & degradation	[57,58]
	*L. delbrueckii*	Adsorption	[59]
	*L. fermentum*	—	[60]
Enterococcaceae	*E. faecium*	—	[61]
Enterobacteriaceae	*E. coli*	Degradation	[62]
*Tetragenococcus halophilus*		Degradation	[63]
Pseudomonadaceae	*P. aeruginosa*	Degradation	[64]
	*P. putida*	Degradation	[65,66]
	*P. stutzeri*	Degradation	[64]
*Xanthomonadaceae*		Degradation	[67]
*Burkholderiaceae*		—	[68]
Corynebacteriaceae	*C. rubrum*	Degradation	[69]
Mycobacteriaceae	*M. fluoranthenivorans*	Degradation	[70]
Nocardiaceae	*N. corynebacterioides*	Degradation	[71,72]
Streptomycetaceae	*S. roseolu*	Degradation	[73]
Bifidobacteriaceae	*B. lactis*	Adsorption	[74]
Flavobacteriaceae	*F. aurantiacum*	Degradation	[75]
*Saccharomyces*	*S. cerevisiae*	Adsorption & degradation	[76]
*Myxomycophyta*	*M. fulvus*	Degradation	[77,78,79]
*Aspergillus niger*		Degradation	[80]
*Candida versatilis*		Degradation	[81]
*Rhizopus oligosporus*		Degradation	[82]
*Pichia occidentalis*		Adsorption & degradation	[83]
*Candida sorboxylosa*		Adsorption & degradation	[83]
*Hanseniaspora opuntiae*		Adsorption & degradation	[83]
*Trametes versicolor*		Degradation	[84]
*White* *-rot fungus Cerrena unicolor*		Degradation	[85]

a This symbol indicates unknown or not mentioned.

**Table 3:** AF-degrading enzymes and their sources.

Degrading Enzyme		Source	Refs.
Intracellular:	Aflatoxin oxidase (AFO)	*Armillariella tabescens*	[104,105]
Extracellular:	Laccase	*White rot fungi*	[106]
	Peroxidase	*Pseudomonas* sp.	[107]
	Reductase	*Mycobacterium smegmatis*	[108]
	Lactoperoxidase	*–*	[109]
	Manganese peroxidase	*Pleurotus ostreatus*	[110]
	Myxobacteria AF degradation enzyme	*Myxococcus fulvus*	[111]

**Table 4:** Microbial localization of AF-degrading substances and degradation products.

Microorganism	AFs	Clearance Rate (%)	Degradation Substances ^a^	Product	Refs.
*Bacillus velezensis DY3108*	B_1_	94.70	Extracellular protein or enzyme	New substances with significantly reduced cytotoxicity	[125]
*Bacillus subtilis UTBSP1*	B_1_	~100	Surfactin and fengycin homologues	– ^b^	[49]
*Bacillus subtilis ANSB060*	M_1_ G_1_ B_1_	60 80.7 81.5	Culture supernatant	–	[50]
*Bacillus pumilus E-1-1-1*	M_1_	89.55	Culture supernatant	–	[52]
*Lysinibacillus fusiformis*	B_1_	61.3	Intracellular protein	New substances with significantly reduced cytotoxicity	[54]
*Sporosarcina* sp.	B_1_	46.9	Intracellular protein	New substances with significantly reduced cytotoxicity	[54]
*Staphylococcus warneri*	B_1_	47.4	Intracellular protein	New substances with significantly reduced cytotoxicity	[54]
*Escherichia coli CG1061*	B_1_	93.7	Intracellular heat-resistant protein	C_16_H_14_ O_5_ and new substances with significantly reduced cytotoxicity	[62]
*Tetragenococcus halophilus CGMCC 3792*	B_1_	66	Viable cells and intracellular active ingredient	C_14_H_20_O_2_	[63]
*Pseudomonas aeruginosa*	*B_1_* *B_2_* *M_1_*	82.8 46.8 31.9	Culture supernatant	New substances	[64]
*Pseudomonas putida* *MTCC 1274 and 2445*	*B_1_*	~90	Culture supernatant	AFD_1_ AFD_2_ AFD_3_	[125]
*Pseudomonas putida*	*B_1_*	80	Culture supernatant and cell lysate	–	[65]
*Stenotrophomonas* sp. *CW117*	B_1_	~100	Culture supernatant	Phthalic anhydride (C_8_H_4_O_3_)	[68]
*Burkholderia* sp. *strain XHY-12*	B_1_ B_2_	>85	–	–	[69]
*Rhodococcus erythropolis*	*B_1_*	100	Extracellular enzymes	–	[126,127]
*Aspergillus niger*	B_1_	58.2	Extracellular enzymes	–	[81]
*Candida versatilis* *CGMCC 3790*	B_1_	69.4	Viable cells and intracellular enzymes	C_14_H_10_O_4_ C_14_H_12_O_3_ C_13_H_12_O_2_ C_11_H_10_O_4_	[82]

a The main location of the degradable substances. b This symbol indicates unknown or not mentioned.

**Table 5:** Detoxification effects of probiotic compounds on AFs.

Probiotics	Degradation Rate (%)	Source	Reaction Conditions	AFs	Refs.
*Lactobacillus bulgaricus,* *L. rhamnosus,* *Bifidobacterium lactis*	38	UHT milk	Incubation with heat-killed bacterial cells (1010 cells/mL) at 4 or 37 °C for 15 min	M_1_	[132]
*Saccharomyces cerevisiae,* *L. plantarum NRRLB-4496,* *L. helveticus ATCC 12046,* *L. lactis JF 3102*	100	Milk	Incubation with heat-killed yeast and/or bacterial cells (107–1010 cells/mL) at room temperature for 1 h	M_1_	[133]
*Streptococcus thermophilus,* *Bifidobacterium bifidum,* *Saccharomyces cerevisiae,* *Kluyveromyces lactis*	94	Baby food	Incubation with 0.5 mL of probiotic mix and 0.5 mL yeast mix for 3 d	B_1_ B_2_	[134]
*Bacillus subtilis,* *Lactobacillus casei,* *Candida utilis*	45.49	– ^a^	–	B_1_	[135]
*Pichia occidentalis,* *Candida sorboxylosa,* *Hanseniaspora opuntiae*	97	Kombucha	Incubation with 200 mL of mother liquor and 10% fermentation broth at 25 °C for 7 d	B_1_	[128]

a Unknown or not mentioned by the authors.

## Data Availability

Data is contained within the article.
